# Quantifying the Intensity of Online Social Support via Large Language Model–Based Evidence Extraction: Development and Validation Study

**DOI:** 10.2196/95025

**Published:** 2026-08-12

**Authors:** Dahyeon Park, Daejin Choi

**Affiliations:** 1 Department of Mathematics, Incheon National University, Incheon, Republic of Korea; 2 College of Artificial Intelligence, Ewha Womans University, 52 Ewhayeodae-gil, Seodaemun-gu, Seoul, 03760, Republic of Korea, 82 2-3277-5947

**Keywords:** online social support, support prediction, AI, large language model, explainable AI, evidence support

## Abstract

**Background:**

Online social support, the interaction among individuals in which one helps another during difficult situations through online platforms such as online forums or social media, has proliferated as a vital tool for personal mental health care. Despite the growing usage and importance of online social support, prior studies have mainly focused on either understanding the characteristics of support seekers or merely identifying types of support, which leaves room for improvement in 2 key areas. First, the intensity of support, indicating the strength of willingness provided by supporters, has been studied little. Second, existing models rely heavily either on handcrafted features or on simple text embeddings of the entire message, which, respectively, fail to capture comprehensive textual features or to provide explainability in classification model decisions.

**Objective:**

This study proposes a deep learning model that not only predicts the intensity of online social support but also provides human-readable evidence that explains the reasoning behind the decision made by the proposed model. While prior studies mainly concentrated on the task of identifying support types, this research quantifies the strength of support, which may better explain how strongly supporters seek to deliver informational and emotional support.

**Methods:**

The proposed model categorizes informational and emotional support into 3 levels—strong, moderate, and weak. It collaborates with a large language model (LLM) to extract key sentences or phrases expressing supportive intent and to compute sentiment scores from both the original post and the reply. All extracted texts are then encoded as a vector representation whose elements indicate the presence of text segments corresponding to predefined features. All the computed features are finally fed into the neural network layers for intensity classification. The proposed model was evaluated on 2 independent, human-annotated datasets collected from previous studies, which consist of 1000 and 400 pairs of support-seeking posts and their associated supporting comments, respectively.

**Results:**

The model achieved superior accuracy compared to baselines on both datasets (0.728/0.694 and 0.709/0.598 for informational support and emotional support, respectively). Through a case study of representative examples, we show that the extracted texts serve as useful evidence explaining the decisions made by the proposed model. For example, explicit intent to support (eg, providing a detailed solution or showing strong empathy) is successfully extracted and used to detect strong support, whereas only indirect support (eg, telling personal stories or offering general advice) is present and can be captured as weak support.

**Conclusions:**

Through cooperation with LLMs, quantifying the intensity of online social support is highly achievable. In addition, the sentences extracted by LLMs can be used to explain the decisions made by the proposed model.

## Introduction

Online social support, an interaction among individuals in which people help one another during difficult situations through online platforms such as online forums or social media [1], has become a key vector for caring for personal mental health in recent years. By providing spaces where users can share experiences, seek advice, and offer empathy anonymously, online digital health platforms such as TalkLife or 7Cups have facilitated the proliferation of online social support for diverse health issues, including HIV, cancer, sexual abuse, and mental health [2].

The increasing popularity of online social support has spurred the research community to understand and predict its characteristics and effects. In particular, focusing on the support seekers, prior studies have primarily analyzed the characteristics of support-seeking messages and the psychological effects of online social support [3,4]. On the other hand, there have been efforts to explore what online social support is delivered and how it is delivered [2,5]. With significant advances in machine learning techniques and the insights provided by prior studies, a few attempts to develop machine learning models for diverse tasks related to online social support, including the classification of support types [6] and the degree of satisfaction with the given support [7], have emerged.

Although these prior studies have provided important insights into understanding and predicting online social support, there is room to improve prior research from 2 perspectives. First, the *intensity of support*, which indicates the strength of willingness provided by supporters, has been little explored, whereas support types (eg, informational or emotional) and their effects on support seekers have been mostly investigated. Since not only the types of online social support but also the strength of support may affect the mental state of support seekers, we believe that predicting and understanding the intensity of online social support are important. Second, the models proposed in previous research have relied either on handcrafted features or on simple text embeddings of the entire support message, which, respectively, make it difficult to capture comprehensive textual features or to provide explainability in classification model decisions.

In this paper, we propose a deep learning–based model that not only predicts the intensity of online social support but also provides human-readable evidence that can show the reason for the decision made by the proposed model. To the best of our knowledge, this is among the first studies to develop a classification model for the intensity of social support. To this end, the proposed model classifies the intensity of informational support (IS) and emotional support (ES) for a given supporting message into 3 classes (strong, moderate, and weak). The proposed model extracts important sentences or phrases that may indicate the specific intent of support (eg, showing empathy or providing solutions) by leveraging a large language model (LLM), which has shown great success in diverse natural language processing (NLP) tasks. The final decision (ie, intensity of online social support) is made by using the feature vectors encoded from the extracted text. In addition, the extracted text is presented alongside the intensity of social support to provide human-readable evidence. Our evaluation on 2 human-annotated datasets has demonstrated that the proposed model can accurately estimate the strength of IS and ES; the accuracy values for IS and ES on the 2 datasets are 0.728 (0.709) and 0.694 (0.598), which outperform all other baseline models. In addition, through a case study on representative examples, we reveal that the extracted text can be used as useful evidence that explains the decisions made by the proposed model—we find that the explicit intent of support (eg, providing a detailed solution or showing strong empathy) is successfully extracted and used to detect strong support, while only indirect support (eg, telling personal stories or offering general advice) is present and can be captured in weak support.

## Methods

### Prediction Model

#### Task Definition

In this study, we propose a deep learning model to classify the intensities of ES and IS, which indicate how strongly these forms of support are represented in a given comment. To this end, we define the prediction task as a multiclass classification problem with 3 labels: strong, moderate, and weak. Note that the intensities of ES and IS are predicted by 2 different models, each designed for the corresponding intensity estimation.

#### Overall Architecture

**Figure 1.**
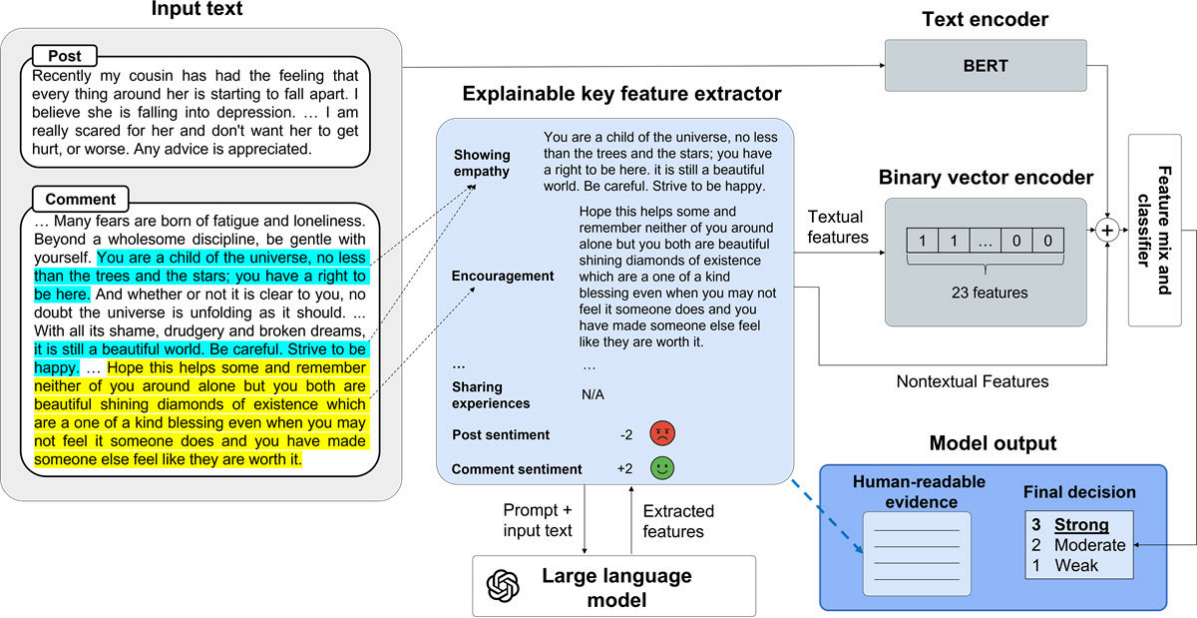
The overall architecture of the proposed model for estimating the intensity of emotional or informational support. The input text consists of a support-seeking post and its associated comment for support. The first component, the explainable key feature extractor, interacts with a large language model (LLM) to (1) extract the key parts of the text related to each predefined factor and (2) compute the sentiment intensity for the given post and comment in the range of −2 (ie, strong negative) to 2 (strong positive). The extracted texts are fed into the binary vector encoder, which outputs a binary vector in which each element indicates the presence of a predefined feature. All the concatenated feature vectors from the encoders are then forwarded to the final classifier layer, which determines the intensity of the support as strong, moderate, or weak. Note that all outputs of the key feature extractor (ie, extracted text segments and sentiment scores) can be used as human-readable evidence supporting the final decision. BERT: bidirectional encoder representations from transformers; N/A: not available.

The overview of the proposed model is illustrated in Figure 1. The model consists of four main components: (1) *explainable key feature extractor*, which communicates with an LLM to extract key textual features and compute sentiment scores for a post or comment; (2) *text encoder* (TE), which encodes the input post and its associated comment into vector representations; (3) *binary vector encoder* (BVE), which encodes the extracted texts into a vector representation in which each element indicates the existence of a text segment corresponding to a predefined feature; and (4) *intensity classifier*, which combines all the outputs of the encoders and the key feature extractor to finally predict the intensity of ES and IS.

#### Explainable Key Feature Extractor

One of the key characteristics of the proposed model is its ability to extract key textual parts from posts and comments across diverse perspectives and to compute sentiment scores, which are used not only to identify the intensity of online social support but also as human-readable evidence. There are several rationales for this approach. First, it has been reported that IS or ES tends to include specific factors for effective support, such as personal experiences, reflections of feelings, expressions of empathy, or informational solutions [8]. Therefore, we design the prediction model to focus more on the important cues for predicting the intensity of support. Second, although neural network–based models have shown great success across many tasks in diverse domains, many models suffer from a lack of interpretability that can reveal the reasons for decisions of the model. In recent years, a few attempts have been made to provide evidence for the output of the model to explain the results, which can give not only a better understanding of the process of the model but also great insights into the target task. Since the texts extracted and used by the proposed model are human-readable, they can also be used for the interpretation of the results by the proposed model, which enhances the explainability of the proposed model.

Using these rationales, we design the feature extractor component to communicate with an LLM that has shown great success in diverse NLP tasks. In particular, the feature extractor requires key sentences and phrases that indicate specific expressions related to IS or ES, which include empathy, encouragement, the sharing of personal experiences, the suggestion of solutions, and the sharing of external resources or references. The LLM is also responsible for computing sentiment scores for both the post and the comment in the range of −2 (“strong negative”) to 2 (“strong positive”). All the features, their output types, and the prompts used to communicate with the LLM are summarized in Table 1 and Multimedia Appendix 1.

**Table 1.** The key features and their output types^a^.

Type	Model	Output type	IS^b^/ES^c^/both
Advice request in post	An explicit existence of a request for advice in a given support-seeking post.	Binary	Both
External link or reference	The texts of the external references or URLs.	Text	Both
Informational answering	The sentences or paragraphs that indicate advice, recommendations, or suggestions.	Text	Both
Sharing experience	The sentences or paragraphs to share personal experiences or the experiences of others.	Text	Both
Showing empathy	The sentences or paragraphs that indicate empathy.	Text	Both
Encouragement	The sentences or paragraphs that provide encouragement.	Text	Both
Request to contact	The sentences or paragraphs that indicate explicit request to be contacted or provide a way to initiate contact.	Text	Both
Personal emotion	The sentences or paragraphs that contain nonsupportive emotion. These may include expressions of the speaker’s own emotional state, observations, or personal reflections that are not directed at supporting the other person.	Text	Both
Reflection	The sentences or paragraphs that include reflective language but do not clearly offer emotional support, encouragement, problem-solving advice, or personal experience sharing. These may include vague expressions of empathy, indirect emotional comments, or ambiguous feelings that lack clear supportive intent.	Text	Both
Emotional validation	The sentences or paragraphs that explicitly affirm the legitimacy and normality of the target individual's emotional responses.	Text	Both
Shared emotional experience	The sentences or paragraphs including expressions where the commenter discloses similar personal experiences of distress, fostering a sense of shared suffering and reducing perceived isolation through affective alignment.	Text	Both
Hope expression	The sentences or paragraphs that convey optimism about the target individual's future or recovery, emphasizing positive outlooks and perceived resilience.	Text	Both
Nonjudgmental support	The sentences or paragraphs capturing explicit expressions of nonevaluative stance toward the target individual's situation, choices, or emotions.	Text	Both
Explicit emotion recognition	The sentences or paragraphs that explicitly recognize and label the specific emotions experienced by the target individual, reflecting a higher level of empathic attunement through precise emotional identification.	Text	Both
Sentiment scores	The integer values that represent degrees of the sentiments indicated in the given text. The values are in a range of –2 to +2, where –2 and +2 represent strong negative and positive sentiment, respectively.	Integer	Both
Self-disclosure	The sentences or paragraphs that disclose a past personal experience.	Text	IS
General suggestions	The sentences or paragraphs that offer suggestions. This includes questions, conditional statements, or advice that lacks specificity or clear guidance. These sentences may sound helpful but do not provide concrete or actionable information.	Text	IS
General advice	The sentences or paragraphs that give general advice (eg, life advice or statements applicable to anyone).	Text	IS
Detailed solution	The sentences or paragraphs that clearly provide practical solutions or step-by-step instructions to resolve a problem or situation.	Text	IS
Information sources	The sentences or paragraphs that include concrete references to external resources, such as named websites, books, medical sources, or research-backed information.	Text	IS
Nonemotional expression	The emotionally neutral sentences or paragraphs that usually include information, observation, or general statements.	Text	ES
Strong empathy	The sentences or paragraphs that express strong empathy toward the recipient.	Text	ES
Strong encouragement	The sentences or paragraphs that express strong help or encouragement.	Text	ES

a The last column indicates whether the features are extracted to determine the intensity of IS, ES, or both.

b IS: informational support.

c ES: emotional support.

All the extracted features and the computed sentiment scores follow different paths; the text features are fed into the text encoder component, while the sentiment scores are concatenated with the output of the text feature encoder.

#### Text Encoder

The text encoder is responsible for computing linguistic feature vectors for the given pair consisting of a support-seeking post and its associated supporting comment. To this end, we comprehensively investigated popular pretrained language models (PLMs), including bidirectional encoder representations from transformers (BERT) [9] and RoBERTa (robustly optimized BERT approach) [10], and finally decided to use BERT, which outperformed all the other PLM models. The whole process of calculating the final output (Emb_text_) can be formulated as follows:


(1) $${Emb}_{text}\text{=}BERT\left(post\text{\textbackslash{} }⨁\text{\textbackslash{} }comment\text{\textbackslash{} }\right)\in R^{d}$$

where *d* indicates the dimension of the embedding vector.

#### BVE

The BVE computes a feature vector whose elements indicate whether a text segment exists for each predefined text feature. For example, in Figure 1, 2 text segments are extracted for 2 features, “showing empathy” and “encouragement.” In this case, the 2 elements of the binary vector that correspond to these features are set to 1, while all the other elements are set to zero.

#### Intensity Classifier

The last component, the intensity classifier, concatenates all the encoded feature vectors (ie, text embedding, binary vector, and the sentiment scores) and finally passes the concatenated vector through an activation layer for the final decision (ie, the intensity of IS and ES). Formally, the decision by the classifier can be defined as:


(2) $${\hat{y}}_{i}=F\left({Emb}_{\text{text}}⊕V_{\text{binary}}⊕V_{\text{sentiment}}\right)$$

where *y_i_* ∈ {1,2,3} represents the classes of the intensity of support in the order of weak, moderate, and strong, respectively. Note that *V*_sentiment_ is the sentiment scores of the post and the comment, computed by an LLM from key feature extractor.

To optimize the model parameters, we use categorical cross-entropy loss, a popular loss function for multiclass classification tasks. To address the class imbalance problem, we use inverse ratio–based class weights (CWs) to the loss function. The final formula of the loss function can be written as follows:


(3) $$L=-\sum_{i\text{=}1}^{C}w_{i}y_{i}\log{\hat{p}}_{i}$$

where *C*, *y_i_*, and ${\hat{p}}_{i}$ are the number of classes, the ground-truth label, and the predicted probability for class *i*, computed using the softmax function, respectively. The CW for class *i*, denoted as *w_i_*, is defined as the inverse of the ratio of the number of instances in class *i* over the training set.

### Experimental Setup

#### Datasets

We evaluated the proposed model with 2 independent datasets constructed by Peng et al [11] and Sharma and De Choudhury [12], which consist of 1000 and 400 pairs of support-seeking posts and their associated supporting comments, respectively. The supporting comments in these datasets were inspected and labeled by more than 2 human annotators who were instructed in advance. Note that the detailed methods including data collection, refinement, validation for the data labeling, and agreement among the annotators have been reported by Peng et al [11] and Sharma and De Choudhury [12], respectively. The Cohen κ values for IS and ES in Sharma and De Choudhury [12] are 0.879 and 0.876, respectively, while Peng et al [11] reported Cronbach α values of 0.91 and 0.81 for IS and ES, respectively. We measured the performance of the proposed model for predicting the intensity of social support with 5-fold cross-validation.

#### Hyperparameters and Implementation Details

We conducted a grid search over the following hyperparameter space: epoch ∈ {3, 5, 10}, batch size ∈ {4, 8, 16}, and learning rate ∈ {1e-5, 2e-5, 5e-5}. The optimal hyperparameter configuration was determined based on the average performance obtained from 5-fold cross-validation. We finally set the batch size, learning rate, and epoch counts to 16, 2e-5, and 5, respectively. After investigating multiple PLMs, we finally decided to use the BERT-base-uncased model to generate feature vectors of the extracted texts. We set different output dimensions of FC_aggr_ for the intensity classification of IS and ES, which are 768 and 1600, respectively. We used the AdamW optimizer [13] and softmax for model training and activation, respectively. Note that all the proposed models were implemented on a machine of Apple M4 Pro CPU (with integrated GPU), 24 GB of RAM, using Python 3.11.11 (Python Software Foundation) and PyTorch 2.2.0 (PyTorch Foundation) on macOS 15.6.1 (Apple).

#### Baseline Models

We selected the following models as baseline models for evaluation:

- LLMs: in recent years, LLMs have shown great inference capabilities across diverse tasks. To measure and compare their ability to perform the classification task with that of the proposed models, we selected 3 well-known LLMs: ChatGPT (GPT-3.5-turbo), ChatGPT (GPT-4), and ChatGPT (GPT-4o).
- PLMs: despite their smaller size in terms of the number of parameters, PLMs such as BERT and RoBERTa are still widely used across diverse NLP tasks. Here, we choose RoBERTa and BERT as the baseline models for comparison.

#### Evaluation Metric

Given the number of TP (true positive), FP (false positive), TN (true negative), and FN (false negative) instances, we evaluated the proposed model by measuring accuracy, precision, recall, and *F*_1_-score, which are defined as follows:

- Accuracy: $\frac{TP\text{+}TN}{TP\text{+}TN\text{+}FP\text{+}FN}$
- Precision: $\frac{\text{TP}}{\text{TP}\text{+}\text{FP}}$
- Recall: $\frac{\text{TP}}{\text{TP}\text{+}\text{FN}}$
- *F*_1_-score: $\frac{2\times \text{Precision}\times \text{Recall}}{\text{Precision}\text{+}\text{Recall}}$

Note that we report the average scores of 4 metrics across the 5-fold cross-validation. To assess the statistical significance across different trials, we conducted all experiments twice and measured *P* values from a paired *t* test between 2 trials. All the *P* values from the paired *t* test across multiple experiments are greater than 0.05, indicating no significant differences among the trials.

### Ethical Considerations

Institutional review board review was not required for this study under local regulations and institutional policies. According to Article 13 of the Enforcement Regulations of the Bioethics and Safety Act of South Korea [14], research that exclusively uses publicly available or secondary data and does not collect, store, or record any personally identifiable information is not subject to formal ethics board review. This investigation consists solely of a secondary analysis of 2 independent, fully anonymized, and human-annotated datasets from previous studies (comprising 1000 and 400 text pairs, respectively). The authors had no direct interaction with human participants or any intervention involving them, and no personal data were accessed. Therefore, this study poses zero risk to participants and meets the criteria for exemption from formal ethical review.

## Results

### Overall Performance

Tables 2 and 3 present the evaluation results of the baseline and proposed models that identify the intensity of IS and ES, respectively, using 2 public datasets. Note that we also report the performance of 2 more variants (TE+BVE and TE+BE+CW) of the proposed model for an ablation study, which are the models with (1) only TE and BVE and (2) a class-weighted loss function, respectively. As shown in both tables, the proposed model outperforms all other baseline models on the dataset by Peng et al [11]. The accuracy and *F*_1_-scores of the proposed model (all-inclusive) for IS and ES are 0.728 (0.709) and 0.730 (0.709), respectively, demonstrating that the proposed model can accurately estimate the intensity of these support systems.

**Table 2.** Evaluation results of the baseline and proposed models for informational support on 2 datasets^a^.

Type and model	Peng et al [11]				Sharma and De Choudhury [12]			
	Accuracy	Precision	Recall	*F*_1_-score	Accuracy	Precision	Recall	*F*_1_-score
LLM^b^								
GPT-3.5-turbo	0.469	0.586	0.412	0.395^c^	0.273	0.505	0.385	0.237^c^
GPT-4	0.487	0.542	0.477	0.468^d^	0.389	0.486	0.466	0.381^c^
GPT-4o	0.487	0.511	0.492	0.490^c^	0.626	0.661	0.630	0.627^e^
PLM^f^								
RoBERTa^g^	0.620	0.621	0.650	0.630^e^	0.625	0.612	0.577	0.555^d^
BERT^h^	0.673	0.677	0.681	0.679^e^	0.675	0.663	0.636	0.641^e^
Proposed (TE^i^+BVE^j^)	0.693	0.700	0.700	0.700	0.675	0.637	0.623	0.627
Proposed (TE+BVE+CW^k^)	0.711	0.721	0.716	0.716	0.692	0.686	0.666	0.671
Proposed (all inclusive)	0.728	0.739	0.726	0.730	0.694	0.694	0.675	0.680

a Average values of the performance metrics across the 5-fold cross-validation are reported. A paired *t* test was conducted between the proposed model and the baseline models for *F*_1_-scores across the 5-fold cross-validation (*P*<.05,* P*<.01, *P*<.001).

b LLM: large language models.

c *P*<.001.

d *P*<.01.

e *P*<.05.

f PLM: pretrained language models.

g RoBERTa: Robustly Optimized BERT approach.

h BERT: bidirectional encoder representations from transformers.

i TE: text encoder.

j BVE: binary vector encoder.

k CW: class weight.

**Table 3.** Evaluation results of the baseline and proposed models for emotional support on 2 datasets^a^.

Type and model	Peng et al [11]				Sharma and De Choudhury [12]			
	Accuracy	Precision	Recall	*F*_1_-score	Accuracy	Precision	Recall	*F*_1_-score
LLM^b^								
GPT-3.5-turbo	0.531	0.534	0.501	0.509^c^	0.447	0.534	0.455	0.367^c^
GPT-4	0.451	0.472	0.474	0.455^c^	0.523	0.541	0.528	0.500^c^
GPT-4o	0.496	0.500	0.527	0.499^c^	0.551	0.587	0.552	0.555^c^
PLM^d^								
RoBERTa	0.664	0.683	0.642	0.645^e^	0.625	0.602	0.627	0.605^e^
BERT^f^	0.664	0.714	0.604	0.626^e^	0.613	0.623	0.612	0.612^e^
Proposed (TE^g^+BVE^h^)	0.667	0.700	0.640	0.657	0.625	0.629	0.625	0.627
Proposed (TE+BVE+CW^i^)	0.686	0.707	0.671	0.682	0.629	0.634	0.630	0.627
Proposed (all inclusive)	0.709	0.731	0.709	0.709	0.598	0.616	0.600	0.596

a Average values of the performance metrics across the 5-fold cross-validation are reported. A paired *t* test was conducted between the proposed model and the baseline models for *F*_1_-scores across the 5-fold cross-validation (*P*<.05, *P*<.01, *P*<.001).

b LLM: large language models.

c *P*<.001.

d PLM: pretrained language models.

e *P*<.05.

f BERT: bidirectional encoder representations from transformers.

g TE: text encoder.

h BVE: binary vector encoder.

i CW: class weight.

When we look at the performance of the baseline models, more recent versions of LLMs indicate improved performance in IS; the *F*_1_-scores for ChatGPT (GPT-3.5-turbo), ChatGPT (GPT-4), and ChatGPT (GPT-4o) on the dataset of Peng et al [11] are 0.395, 0.468, and 0.490, respectively, while those on the dataset of Sharma and De Choudhury [12] are 0.237, 0.381, and 0.627, respectively. These results imply that more recent versions of LLMs have greater capability to determine the intensity of IS. Interestingly, despite the much larger number of model parameters, the performance of LLMs is generally worse than that of popular PLMs, BERT and RoBERTa, in both IS and ES; the *F*_1_-scores of BERT and RoBERTa are higher than those of LLMs in all cases except one, in which GPT-4o shows improved performance over RoBERTa for IS classification. These results show that fine-tuning PLMs is generally more suitable than solely using LLMs.

Tables 2 and 3 also show that the primary components of the proposed model contribute to model performance. When we compared the performance of the proposed model across different versions, the performance improved in the order of the all-inclusive, without Meta FE, and Text FE cases. The *F*_1_-scores for IS and ES cases on the dataset of Peng et al [11] are 0.700 (0.657), 0.716 (0.682), and 0.730 (0.709), indicating that the feature extractors play a role in identifying the intensity of social support to some extent.

**Figure 2.**
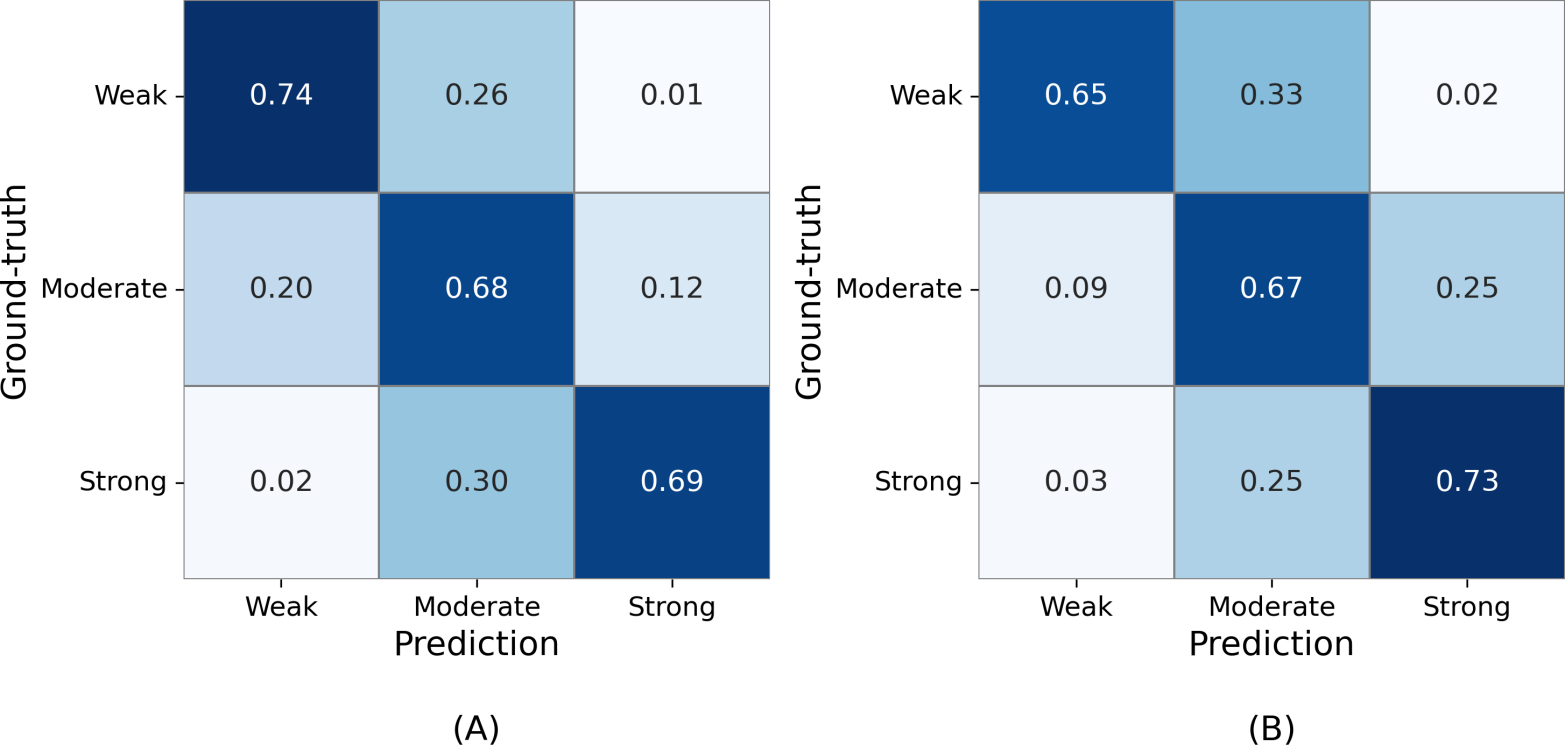
The heatmaps for the predicted and actual classes are depicted: (A) informational support scores and (B) emotional support scores.

We next compared the predicted and actual classes to investigate how close the predicted results are to the actual classes for both datasets, as visualized in the heatmaps in Figure 2. Note that we normalized the values along the horizontal line (ie, the sum of the values in a row is 1), and darker cells indicate more instances. As shown in Figure 2, the diagonal cells are the darkest, indicating that the proposed model can accurately distinguish among strong and weak informational and emotional social support. In addition, the cells near the diagonal are darker than the other cells, and the extremely incorrect cases (ie, lower-left and upper-right cells) are nearly zero, demonstrating that the incorrect instances are close to the actual classes.

### Case Study for Evidence Support

The proposed model in this study leverages explainability to interpret classification results. To illustrate this, a case study was conducted comparing the decision results with the extracted features provided for each decision.

**Figure 3.**
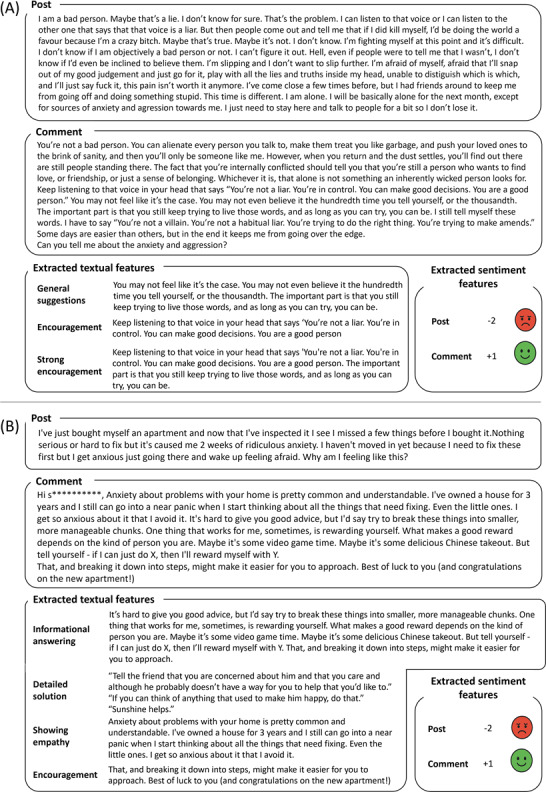
The extracted features for 2 different cases are shown. The informational support and emotional support labels of the 2 cases are weak/strong (A) and strong/weak (B), respectively.

Figure 3 shows 2 case examples where the predicted intensities of IS and ES are correct. Here, we sample pairs consisting of a post and a comment whose predicted intensities of support differ; the intensity classes of IS and ES in the first case are weak and strong, respectively, and vice versa for the second case. In both cases, the phrases or sentences associated with the predefined features, such as “Informational Answering,” “General Suggestions,” “Strong Encouragement,” and “Detailed Solutions,” seem to be reasonably extracted from the supporting comments. For example, the sentence “Anxiety about problems with your home is pretty common and understandable.” is captured by the feature “Showing empathy,” demonstrating that the LLM-cooperative feature extractor can extract key parts of the texts to determine the intensity of social support.

In addition, Figure 3 provides insight into the decisions made by the proposed model. That is, in the first case, the only feature related to IS is “General Suggestions,” whereas in the second case they are “Informational Answering” and “Detailed Solution.” Moreover, the text of “General Suggestions” in the first case is generic and unlikely to be related to the specific situation of support seekers, and it could also be used in other situations. On the other hand, the “Detailed Solution” in the second example offers guidance to support seekers on what to do (eg, “Tell the friend that you are concerned about him...”). Since the solution explicitly provides IS, the proposed model decided the IS of the given support was strong.

Similarly, a comparison of the intensity classes of ES and the extracted texts for the 2 cases explains the reasons for the decisions made by the proposed model. That is, while the support in the first case includes text of “Strong Encouragement” (ie, “The important part is that you still keep trying to live those words, and as long as you can try, you can be.”), only weak empathy is represented in the second one. These results show the explainability of the proposed model—the explicit intent of support (eg, providing a detailed solution or showing strong empathy) is successfully captured and used to detect strong support, whereas only implicit support (eg, telling personal stories or offering general advice) is observed in weak support.

## Discussion

In this study, we propose an explainable deep learning model that estimates the intensity of IS and ES. We now discuss the implications and limitations of this work.

### LLM-Cooperative Model to Classify the Intensity of Social Support

LLMs have shown great success in diverse tasks. However, we design an LLM-cooperative model that communicates with an LLM to extract key sentences and phrases instead of using an LLM as a direct predictor for quantifying the intensity of social support. The rationale for the design comes from the characteristics of the decision process for the given task. That is, to determine the intensity of social support, it is crucial to concentrate more on the key parts that reflect the supporter’s intent, such as empathy or advice, since both the support-seeking post and the supporting comment tend to be long and descriptive. Therefore, when an LLM is used directly for this task, the entire process may be conducted implicitly and be vulnerable to noise that is irrelevant to the classification. In contrast, the proposed model explicitly extracts the parts of the supporting text related to the supporter’s intent and makes its decision based on the extracted features. As shown in the “Results” section, our evaluation demonstrates that the proposed model outperforms all recent LLMs, which implies that the explicit architecture of the proposed model leverages its ability to estimate the intensity of social support.

### Evidence Supporting Improved Explainability

In recent years, deep learning models have been applied to diverse tasks with remarkable success. Despite the strong performance, their inherent “black box” nature—an inability to explain the reasoning behind their decisions—risks limiting their general applicability and feasibility. To address this, we first extract the partial texts that contain supportive intent from a supporter, which are crucial for determining the intensity of IS and ES. Based on this, the proposed model then conducts the classification task using a vector representation of the presence of the extracted texts. Here, the extracted texts can be used as human-readable evidence for the model’s decisions, thereby leveraging the explainability of the proposed model. We believe that enhanced explainability of the proposed model will improve its feasibility.

### Limitations

Despite the contributions of the proposed model that can accurately determine the intensity of IS and ES with explainable evidence, we clearly acknowledge the limitations of this study. First, the intensity of social support in this study was measured and labeled from experts’ perspectives, which may not directly reflect the actual effectiveness experienced by support seekers. Thus, even though the intensity of social support is important for understanding what resources are provided to support seekers and how, future research should investigate the relationship between support intensity and its direct psychological, behavioral, or clinical outcomes on support seekers. Second, the proposed model was evaluated on 2 human-labeled datasets. Thus, we caution against generalizing the methodology and findings to other languages or platforms. Lastly, while the model’s interpretability was illustrated through case studies, a rigorous quantitative evaluation of the extracted rationales (eg, through human assessment or expert ratings) is required to fully validate its explainability. We plan to address these dimensions in future work.

### Comparison With Prior Work

#### Analysis of Online Social Support

With the increasing use of online platforms, online social support, in which support seekers and supporters interact within online communities, has become prevalent across diverse topics, including cancer [15-17], HIV [11], pregnancy [18], and sexual experiences [19,20], on multiple platforms such as Reddit (Reddit, Inc) [21] and TalkLife (TalkLife Ltd) [22]. Such popularity has, in turn, attracted the research community to investigate online social support for mental health. Prior studies have mainly focused on support seekers, particularly the behavioral traits of support seekers [21,22] or the content characteristics of support-seeking posts [23,24]. For example, Andalibi et al [21] characterized the support-seeking behaviors of users with a history of sexual abuse on Reddit, while Kushner and Sharma [22] analyzed temporal patterns of supporting activities in TalkLife, revealing that the intensity of online social support activity varies over time. Undoubtedly, there has been much investigation into the effects of online social support from psychological or behavioral perspectives [25-29]. By analyzing the language of social support in mental health subreddits on Reddit, De Choudhury and Kiciman [27] demonstrated that online social support can reduce the risk of individuals’ suicidal ideation, while Pruksachatkun et al [4] found that social support affects support seekers by changing individuals’ cognitive status.

In recent years, there has been emerging work that seeks to understand the behavioral and content characteristics of supporters and their messages. Leveraging the theoretical definitions of supportive messages [30-32], there have been a few attempts to characterize types of online social support [31,33,34]. One popular classification scheme was suggested by Goldsmith et al [34], which divides online social support into 2 categories: informational and emotional. Based on the classification scheme, the properties and characteristics of supportive messages have been investigated. For example, Wang et al [35] found that IS responses tended to exhibit higher lexical alignment by reusing specific terms and concepts mentioned in the original question, while ES responses showed stronger syntactic alignment by mirroring the sentence structures and expressive styles of the questioner to convey empathy. Coursaris and Liu [5] analyzed the differences between IS and ES and found that support provided by women tends to be more emotional and shorter. By investigating ES in Seva—an addiction recovery app based on online social support, Yang et al [36] demonstrated that stronger emotional expression can help the recovery from mental illness among support seekers. Peng et al [11] explored the relationships between IS and ES and the satisfaction of the support seekers and found that IS is more strongly associated with higher user satisfaction.

#### Predicting Online Social Support

The great insights into types of online social support and their characteristics, provided by prior work, have attracted the research community to develop machine learning models to distinguish among types of online social support. For example, Deetjen and Powell [37] developed a Bayesian classifier to distinguish between IS and ES based on linguistic patterns indicated in support posts for diabetes, hypertension, and fibromyalgia, while Huang et al [38] proposed a Naive Bayes classifier for the same task but on different types of diseases, including breast and prostate cancer. Biyani et al [6] used 62 features, including vocabulary or orthographic characteristics, LIWC (Linguistic Inquiry and Word Count)-based emotional vocabulary (Pennebaker Conglomerates, Inc), and structural or interaction indicators, to classify ES and IS. Alrashidi et al [39] slightly reframed the task as a multilabel classification task, which assigns 5 independent labels for a given support post: emotional, informational, tangible, esteem, and network support. Jozani et al [40] attempted to classify ES and IS with a multimodal deep learning model using not only text representations of Q&A pairs but also behavioral signals, such as response latency, user interaction patterns, and profile information.

Despite the great contributions of these studies, most studies have focused on understanding support seekers or predicting the types of social support, with little attention to estimating the intensity of social support, which is the target task of this study. In addition, the proposed model can provide evidence that demonstrates the reasons for the decisions, which mitigates the lack of explainability inherent in the black-box architecture of deep learning models.

### Conclusions

In this study, we proposed a deep learning model that classifies the intensity of IS and ES into 3 levels. Through cooperation with an LLM, the proposed model extracts key textual cues and sentiment scores, which are used to determine the intensity of social support. Our evaluation with 2 human-annotated datasets demonstrates superior predictive performance compared with baseline models. In addition, we conducted a qualitative case study that illustrates how human-interpretable evidence extracted by LLMs can be leveraged to enhance the explainability of the proposed model. We believe that the proposed work in this study contributed to a more comprehensive understanding of online social interactions and suggested a promising direction for applying explainable AI methods to support timely interventions in the field of mental health and well-being.

## Supplementary material

10.2196/95025 Multimedia Appendix 1 The prompts used for extracting all the features.

## Abbreviations

BERT: bidirectional encoder representations from transformers
BVE: binary vector encoder
CW: class weight
ES: emotional support
FN: false negative
FP: false positive
IS: informational support
LIWC: Linguistic Inquiry and Word Count
LLM: large language model
NLP: natural language processing
PLM: pretrained language model
RoBERTa: robustly optimized BERT approach
TE: text encoder
TN: true negative
TP: true positive

## References

[1] Wright KB, Bell SB, Wright KB, Bell SB (2003). Health-related support groups on the internet: linking empirical findings to social support and computer-mediated communication theory. J Health Psychol.

[2] Kim M, Saha K, De Choudhury M, Choi D (2023). Supporters first: understanding online social support on mental health from a supporter perspective. Proc ACM Hum-Comput Interact.

[3] Saha K, Ernala SK, Dutta S, Sharma E, De Choudhury M, Meiselwitz G (2020). Social Computing and Social Media Participation, User Experience, Consumer Experience, and Applications of Social Computing. 12th International Conference, SCSM 2020, Held as Part of the 22nd HCI International Conference, HCII 2020, Copenhagen, Denmark, July 19–24, 2020, Proceedings, Part II.

[4] Pruksachatkun Y, Pendse SR, Sharma A (2019). Proceedings of the 2019 CHI Conference on Human Factors in Computing Systems.

[5] Coursaris CK, Liu M (2009). An analysis of social support exchanges in online HIV/AIDS self-help groups. Comput Human Behav.

[6] Biyani P, Caragea C, Mitra P, Yen J, Tsujii J, Hajic J (2014). Proceedings of COLING 2014, the 25th International Conference on Computational Linguistics: Technical Papers.

[7] Karusala N, Seeh DO, Mugo C (2021). “That courage to encourage”: participation and aspirations in chat-based peer support for youth living with HIV. Proc CHI Conf Hum Factors Comput Syst.

[8] McIver JP, Carmines EG (1981). Unidimensional Scaling.

[9] Devlin J, Chang MW, Lee K, Toutanova K, Burstein J, Doran C, Solorio T (2019). Proceedings of the 2019 Conference of the North American Chapter of the Association for Computational Linguistics: Human Language Technologies, Volume 1 (Long and Short Papers).

[10] Liu Y, Ott M, Goyal N, Du J (2019). RoBERTa: a robustly optimized BERT pretraining approach. arXiv.

[11] Peng Z, Ma X, Yang D, Tsang KW, Guo Q (2021). Effects of support-seekers’ community knowledge on their expressed satisfaction with the received comments in mental health communities. Proc CHI Conf Hum Factors Comput Syst.

[12] Sharma E, De Choudhury M (2018). Mental health support and its relationship to linguistic accommodation in online communities. Proc CHI Conf Hum Factors Comput Syst.

[13] Loshchilov I, Hutter F Decoupled weight decay regularization. https://openreview.net/pdf?id=Bkg6RiCqY7.

[14] Ministry of Health and Welfare Enforcement rule of the bioethics and safety act, ordinance of the Ministry of Health and Welfare no. 1115, article 13 (promulgated Jun 2, 2025, effective Dec 3, 2025) [Article in Korean]. National Law Information Center.

[15] Smith CE, Levonian Z, Ma H (2020). “I cannot do all of this alone”: exploring instrumental and prayer support in online health communities. ACM Trans Comput-Hum Interact.

[16] Yang D, Kraut RE, Smith T, Mayfield E, Jurafsky D (2019). Proceedings of the 2019 CHI Conference on Human Factors in Computing Systems.

[17] Winzelberg AJ, Classen C, Alpers GW (2003). Evaluation of an internet support group for women with primary breast cancer. Cancer.

[18] Gui X, Chen Y, Kou Y, Pine K, Chen Y (2017). Investigating support seeking from peers for pregnancy in online health communities. Proc ACM Hum-Comput Interact.

[19] Hartikainen H, Razi A, Wisniewski P (2021). Safe sexting: the advice and support adolescents receive from peers regarding online sexual risks. Proc ACM Hum-Comput Interact.

[20] Razi A, Badillo-Urquiola K, Wisniewski PJ (2020). Let’s talk about sext: how adolescents seek support and advice about their online sexual experiences. Proc CHI Conf Hum Factors Comput Syst.

[21] Andalibi N, Haimson OL, De Choudhury M, Forte A (2016). Understanding social media disclosures of sexual abuse through the lenses of support seeking and anonymity. Proc CHI Conf Hum Factors Comput Syst.

[22] Kushner T, Sharma A (2020). Bursts of activity: temporal patterns of help-seeking and support in online mental health forums. Proc Web Conf.

[23] Kruzan KP, Bazarova NN, Whitlock J (2021). Investigating self-injury support solicitations and responses on a mobile peer support application. Proc ACM Hum Comput Interact.

[24] Kummervold PE, Gammon D, Bergvik S, Johnsen JAK, Hasvold T, Rosenvinge JH (2002). Social support in a wired world: use of online mental health forums in Norway. Nord J Psychiatry.

[25] Sharma A, Choudhury M, Althoff T, Sharma A (2020). Engagement patterns of peer-to-peer interactions on mental health platforms. Proc Int AAAI Conf Web Soc Media.

[26] Chen Y, Xu Y, Kitamura Y, Quigley A, Isbister K, Igarashi T (2021). CHI EA ’21: Extended Abstracts of the 2021 CHI Conference on Human Factors in Computing Systems.

[27] De Choudhury M, Kiciman E (2017). The language of social support in social media and its effect on suicidal ideation risk. Proc Int AAAI Conf Web Soc Media.

[28] Saha K, Sharma A (2020). Causal factors of effective psychosocial outcomes in online mental health communities. Proc Int AAAI Conf Web Soc Media.

[29] Zhou J, Saha K, Lopez Carron IM (2022). Veteran critical theory as a lens to understand veterans’ needs and support on social media. Proc ACM Hum-Comput Interact.

[30] Caplan G (1974). Support Systems and Community Mental Health: Lectures on Concept Development.

[31] Caplan G (1981). Mastery of stress: psychosocial aspects. Am J Psychiatry.

[32] Kahn R, Antonucci T, Baltes PB, Brim OG (1980). Life-Span Development and Behavior (Volume 3).

[33] Braithwaite DO, Waldron VR, Finn J (1999). Communication of social support in computer-mediated groups for people with disabilities. Health Commun.

[34] Goldsmith DJ, McDermott VM, Alexander SC (2000). Helpful, supportive and sensitive: measuring the evaluation of enacted social support in personal relationships. J Soc Pers Relat.

[35] Wang YC, Kraut R, Levine JM (2012). To stay or leave? The relationship of emotional and informational support to commitment in online health support groups. Proc ACM Conf Comput Support Coop Work.

[36] Yang EF, Kornfield R, Liu Y (2023). Using machine learning of online expression to explain recovery trajectories: content analytic approach to studying a substance use disorder forum. J Med Internet Res.

[37] Deetjen U, Powell JA (2016). Informational and emotional elements in online support groups: a Bayesian approach to large-scale content analysis. J Am Med Inform Assoc.

[38] Huang KY, Nambisan P, Uzuner Ö, Sabherwal R, Sumner M (2010). Proceedings of the International Conference on Information Systems, {ICIS} 2010, Saint Louis, Missouri, USA, December 12-15, 2010.

[39] Alrashidi M, Selamat A, Ibrahim R, Krejcar O (2023). Social recommendation for social networks using deep learning approach: a systematic review, taxonomy, issues, and future directions. IEEE Access.

[40] Jozani M, Williams JA, Aleroud A, Bhagat S (2025). Emotional and informational dynamics in question-response pairs in online health communities: a multimodal deep learning approach. Inf Syst Front.

