# Supplementary material for: Quantifying the Intensity of Online Social Support via Large Language Model–Based Evidence Extraction: Development and Validation Study
Source: J Med Internet Res. 2026 Aug 12;28:e95025. doi: 10.2196/95025 (PMC13466868; doi:10.2196/95025)
Supplement: Supplementary file 1 [file jmir-v28-e95025-s001.docx]

| **Features** | **Prompt** |
| --- | --- |
| Advice Request in Post | From the given texts, identify whether the writer explicitly asks for advice, recommendations, or practical solutions to a specific issue. Please answer 'YES' if such sentences or paragraphs are found, or 'NO' if they are not. |
| External Link or Reference | From the given texts, extract all sentences or paragraphs that include links, references, or recommendations for external resources. Please answer 'NO' if no such sentences or paragraphs are found. |
| Informational Answering | From the given texts, extract and return all sentences or paragraphs where the author provides advice, recommendations, or suggestions to the reader. Please answer 'NO' if no such sentences or paragraphs are found. |
| Sharing Experience | From the given texts, identify and return all sentences or paragraphs where the writer shares personal experiences or the experiences of others. Please answer 'NO' if no such sentences or paragraphs are found. |
| Showing Empathy | From the given texts, identify and return all sentences or paragraphs where the author expresses empathy or understanding toward the reader's feelings or situation. Please answer 'NO' if no such sentences or paragraphs are found. |
| Encouragement | From the given texts, extract and return all sentences or paragraphs where the author provides encouragement or support to the reader. Please answer 'NO' if no such sentences or paragraphs are found. |
| Request to Contact | From the given texts, identify and return all sentences or paragraphs where the writer explicitly requests to be contacted or provides a way to initiate contact. Please answer 'NO' if no such sentences or paragraphs are found. |
| Personal Emotion | From the given texts, identify and return all sentences or paragraphs that contain language which may initially appear empathetic or emotionally supportive, but in context, do not actually provide comfort, encouragement, or emotional support to the recipient. These may include expressions of the speaker’s own emotional state, observations, or personal reflections that are not directed at supporting the other person. Please answer 'NO' if no such sentences or paragraphs are found. |
| Reflection | From the given texts, identify and return all sentences or paragraphs that include emotional or reflective language but do not clearly offer emotional support, encouragement, problem-solving advice, or personal experience sharing. These may include vague expressions of empathy, indirect emotional comments, or ambiguous feelings that lack clear supportive intent. Please answer 'NO' if no such sentences or paragraphs are found. |

| Emotional Validation | From the given texts, identify and return all sentences or paragraphs where the author explicitly validates or normalizes the reader's feelings or situation, indicating that their emotional response or experience is understandable, reasonable, or expected. These may include phrases like "it makes sense that you feel", "anyone in your situation would", or "your feelings are valid". Please answer 'NO' if no such sentences or paragraphs are found. |
| --- | --- |
| Shared Emotional Experience | From the given texts, identify and return all sentences or paragraphs where the author expresses solidarity or a sense of shared suffering with the reader, indicating they have personally experienced similar pain or difficulties. These may include phrases like "I've been there", "I know what it's like", or "I went through something similar". Please answer 'NO' if no such sentences or paragraphs are found. |
| Hope Expression | From the given texts, identify and return all sentences or paragraphs where the author expresses hope, optimism, or belief in the reader's ability to improve, recover, or overcome their current difficulties. These may include encouragement about the future or expressions of confidence in the reader's resilience. Please answer 'NO' if no such sentences or paragraphs are found. |
| Non-judgmental Support | From the given texts, identify and return all sentences or paragraphs where the author explicitly expresses a non-judgmental stance, acceptance, or understanding toward the reader's situation, choices, or feelings, without implying criticism or blame. Please answer 'NO' if no such sentences or paragraphs are found. |
| Explicit Emotion Recognition | From the given texts, identify and return all sentences or paragraphs where the author explicitly names or acknowledges specific emotions that the reader may be experiencing, such as sadness, loneliness, anxiety, or hopelessness, in a way that shows direct recognition of the reader's emotional state. Please answer 'NO' if no such sentences or paragraphs are found. |
| Sentiment Scores | From the given texts, score the writer’s sentiment on a Likert scale ranging from -2 to +2, where -2 represents strong negative sentiment, -1 represents slightly negative sentiment, 0 represents neutral sentiment, +1 represents slightly positive sentiment, and +2 represents very positive sentiment. Provide the score with the rationales of scoring. Please answer 'NO' when there is no obvious evidence of sentiment in the given texts. |
| Self-Disclosure | From the given texts, identify and return all sentences or paragraphs that describe a past personal experience using simple past tense verbs and do not include any form of advice, suggestions, recommendations, or instructions. Please exclude any sentences that contain modal verbs like 'should', 'could', 'might', or imperative forms. Please answer 'NO' if no such sentences or paragraphs are found. |
| General Suggestions | From the given texts, identify and return all sentences or paragraphs that appear to offer advice or suggestions, but in an indirect, tentative, or non-committal manner. This includes questions, conditional statements, or advice that lacks specificity or clear guidance. These sentences may sound helpful but do not provide concrete or actionable information. Please answer 'NO' if no such sentences or paragraphs are found. |
| General Advice | From the given texts, extract and return all sentences or paragraphs where the speaker gives advice, recommendations, or suggestions that are general or non-specific in nature, rather than directly addressing the writer's unique situation. These may include broad life advice or statements applicable to anyone. Please answer 'NO' if no such sentences or paragraphs are found. |
| Detailed Solution | From the given texts, identify and return all sentences or paragraphs that clearly provide practical solutions or step-by-step instructions to resolve a problem or situation. This includes direct recommendations, explicit procedural steps, or actionable advice aimed at helping the reader take specific action. Please answer 'NO' if no such sentences or paragraphs are found. |
| Information Sources | From the given texts, identify and return all sentences or paragraphs that include concrete references to external resources, such as named websites, books, medical sources, or research-backed information. These references should support the advice or information provided and enhance the credibility or specificity of the support. Please answer 'NO' if no such sentences or paragraphs are found. |
| Non-emotional Expression | From the given texts, extract and return all sentences or paragraphs that are emotionally neutral or detached, where the speaker provides information, observation, or general statements without directly acknowledging, validating, or emotionally engaging with the writer’s situation. These include responses that are impersonal or distanced in tone, even if they seem polite or factual. Please answer 'NO' if no such sentences or paragraphs are found. |
| Strong Empathy | From the given texts, extract and return all sentences or paragraphs that express deep empathy, emotional presence, or strong emotional support toward the recipient. These should include expressions that directly acknowledge the recipient's feelings and offer meaningful comfort, such as "I’m so sorry you experience this” or “You are not alone and I care about you.” Please answer 'NO' if no such sentences or paragraphs are found. |
| Strong Encouragement | From the given texts, extract all sentences or paragraphs that show active emotional support, such as offering to help, showing emotional engagement, or explicitly encouraging the recipient in a personal and meaningful way. These should go beyond polite or general statements, and instead demonstrate personal concern or emotional commitment. Please answer 'NO' if no such sentences or paragraphs are found. |
